# Regulatory Roles of Long Non-Coding RNAs in the Central Nervous System and Associated Neurodegenerative Diseases

**DOI:** 10.3389/fncel.2017.00175

**Published:** 2017-06-30

**Authors:** Zhenzhen Quan, Da Zheng, Hong Qing

**Affiliations:** School of Life Science, Beijing Institute of TechnologyBeijing, China

**Keywords:** long non-coding RNAs (lncRNAs), central nervous system (CNS), neurodegenerative disease, gene expression, transcriptional regulation

## Abstract

Accumulating studies have revealed that the human genome encodes tens of thousands of long non-coding RNAs (lncRNAs), which participate in multiple biological networks modulating gene expression via transcriptional, post-transcriptional and epigenetic regulation. Strikingly, a large fraction of tissue-specific lncRNAs are expressed in the Central Nervous System (CNS) with precisely regulated temporal and spatial expression patterns. These brain-specific lncRNAs are also featured with the cell-type specificity, the highest signals of evolutionary conservation, and their preferential location adjacent to brain-expressed protein-coding genes. Mounting evidence has indicated dysregulation or mutations in lncRNA gene loci are associated with a variety of CNS-associated neurodegenerative disorders, such as Alzheimer’s, Parkinson’s, Huntington’s diseases, Amyotrophic Lateral Sclerosis and others. However, how lncRNAs contribute to these disorders remains to be further explored and studied. In this review article, we systematically and comprehensively summarize the current studies of lncRNAs, demonstrate the specificity of lncRNAs expressed in the brain, their functions during neural development and expression profiles in major cell types of the CNS, highlight the regulatory mechanisms of several studied lncRNAs that may play essential roles in the pathophysiology of neurodegenerative diseases, and discuss the current challenges and future perspectives of lncRNA studies involved in neurodegenerative and other diseases.

## Introduction

For decades, people have considered that “genes and gene-encoded proteins” play crucial roles in regulating diverse cellular processes. However, with the completion of the human genome project, it was observed that less than 5% of the genome is comprised of coding sequences, whereas the majority of human genes are non-protein-coding genes which basically include abundant pseudogenes and comparably numerous non-coding RNAs (ncRNAs; Lander et al., 2001; ENCODE Project Consortium, 2012; FANTOM Consortium and the RIKEN PMI and CLST (DGT), 2014). NcRNAs are broadly defined as all types of RNA that are not translated into proteins due to lack of open reading frames (ORFs). They are also considered to be generated from sections of pseudogenes, which are DNA copies of protein-coding genes with high sequence similarity but have lost at least some of the functions relative to their parental genes over the course of evolution (Milligan and Lipovich, 2014; Ji et al., 2015).

Generally, ncRNAs can be classified into small ncRNAs and long ncRNAs (lncRNAs) based on whether their transcripts are less or larger than 200 nucleotides as a cutoff value (Elling et al., 2016). Small ncRNAs are usually defined as regulatory RNAs with a length ranging from 18 to 35 nucleotides. According to their diverse regulatory functions, small ncRNAs can be divided into several species, including transfer RNAs (tRNAs), ribosomal RNAs (rRNAs), small nuclear RNAs (snRNAs), small nucleolar RNAs (snoRNAs), piwi-interacting RNAs (piRNAs) and endogenous small interfering RNAs (siRNAs), as well as microRNAs (miRNAs; Lander et al., 2001; Costa, 2005; Grivna et al., 2006; Sosinska et al., 2015; Elling et al., 2016). For many short RNAs, their functions have been well-characterized in gene expression control. Apart from these, many of them have been shown to be involved in specific pathologies, including neurodegenerative diseases and cancers. An increasing number of studies have reported that short ncRNAs are involved in Alzheimer’s, Parkinson’s and Huntington’s diseases (AD, PD and HD; Lee et al., 2011; Gstir et al., 2014).

LncRNAs are the largest class of longer (≥200 nt) non-protein coding RNA and their gene number was recently estimated at approximately 9000 within the human genome according to the GENECODE project (ENCODE Project Consortium, 2012). Other lncRNA studies even suggested there are more than 50,000 in the human genome (Managadze et al., 2013). The discovery of large numbers of lncRNAs genes that are redefined as a gene into a transcriptional unit was initially described by the FANTOM Consortium on the mouse transcriptome study (Carninci et al., 2005). Later on, studies of metazoan lncRNA repertoires further demonstrated the ubiquity of lncRNAs, which are however relatively lower-expressed, more tissue-specific and with greater variability from one tissue to another in comparison to protein-coding genes (Derrien et al., 2012; Milligan and Lipovich, 2014). Roles performed by lncRNAs have been evidenced by their participation in multiple networks controlling gene expression in transcriptional, post-transcriptional or epigenetic levels (Batista and Chang, 2013; Kung et al., 2013; Qureshi and Mehler, 2013). However, the biological significance of the majority of lncRNAs is yet to be further elucidated.

It is well known that RNA biology is of foremost significance in the central neural system (CNS) since neural cells are highly transcriptionally active and exhibit a robust expression of ncRNAs (Cherubini et al., 2006; Kapranov et al., 2010; Qureshi and Mehler, 2012). Remarkably, the brain is the organ where a large proportion of tissue-specific lncRNAs are preferentially expressed in particular regions or different cell types (Mercer et al., 2008; Derrien et al., 2012). These lncRNAs in the CNS participate in many aspects of brain functions and their roles in the pathologies of brain-related neurodegenerative diseases have been intensively and comprehensively investigated (Qureshi and Mehler, 2012, 2013). In this review article, we systematically and comprehensively summarize the diverse mechanisms reported for lncRNAs, describe the specificity of lncRNAs expressed in the brain and their functions during neural development as well as their expression profile in major cell types of the CNS. Meanwhile, we present those intensively studied lncRNAs that may play essential roles in the pathophysiology of neurodegenerative diseases, and discuss current challenges and future perspectives of lncRNA studies that are involved in neurodegenerative and other diseases. Hopefully, this review will broaden insights for future research in the field of lncRNAs in the CNS and associated neurodegenerative diseases.

## Characters and Functions of LncRNAs

### Basic Characters, Origins and Categories of LncRNAs

Studies by Derrien T and team (Derrien et al., 2012) aiming at analyzing of lncRNAs from GENCODE V7 catalog revealed that, lncRNAs are produced in a similar way as that of protein-coding genes, whereas they display a striking bias toward two-exon transcripts and they are predominately localized in the chromatin and nucleus, expressed at relatively low levels (Guttman et al., 2009; Quinn and Chang, 2016). In comparison to the protein-coding genes, lncRNAs are under secondary structure conservation, and therefore they are believed to have arisen from different evolutionary pathways (Ponting et al., 2009; Kaessmann, 2010). Yet, origins of lncRNAs are not well understood so far. Due to the fact that lncRNAs harbor low sequence conservations and rapid evolution among mammals, several evolutionary hypotheses could be proposed such as: (1) lncRNAs might be generated by the metamorphosis of protein-coding genes through a gene duplication process; (2) lncRNAs might have evolved from segmental or whole gene duplication of other ncRNA genes; (3) lncRNAs might have originated via *de novo* generation, such as alternations in genomes including chromosomal rearrangement, generation of splice sites and promoters might transform nonfunctional genomic sequences to functional lncRNAs; and (4) transposable elements (TEs) insertions might be another origin of lncRNAs (Ponting et al., 2009; Kaessmann, 2010; Kapusta et al., 2013; Kazemzadeh et al., 2015). However, it was observed that rarely or only a minority (~15%) of lncRNAs showed significant sequence similarity to other lncRNAs or protein-coding genes on positions other than the shared repetitive elements, suggesting that novel lncRNAs genes are basically originated rather from *de novo* non-exonic sequences and/or from TEs than duplication (Derrien et al., 2012; Kapusta et al., 2013).

**Figure 1:**
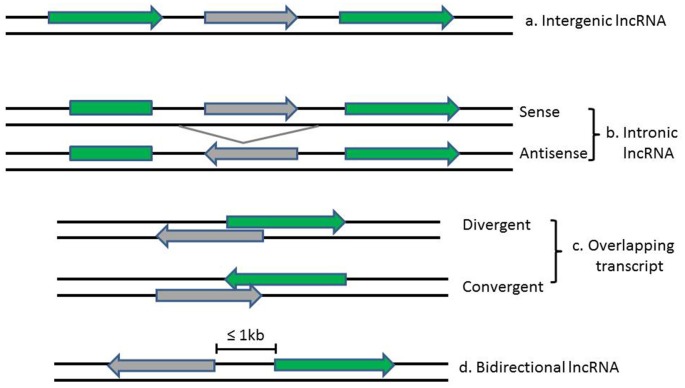
Diagrams show the classification of lncRNAs (in *gray*) according to their position relative to neighboring protein-coding genes (in *green*). Arrows indicate direction of transcription.

LncRNAs and associated lncRNA transcripts have quite heterogeneousgenomic context, regulation, life cycles, mechanism of action and functional profiles. Broadly, lncRNAs can be classified based on their genomic localization and orientation relative to protein coding genes into several categories: (1) long intergenic noncoding RNAs (LincRNAs), consisting of separate transcript units that are located between but do not overlap with protein-coding genes; (2) intronic transcripts, that are located within intron regions of protein-coding genes (sense or antisense); (3) overlapping lncRNAs that are overlapping with other genes either divergently or convergently transcribed; and (4) bidirectional ncRNAs (BincRNAs) with transcripts that are transcribed from divergent bidirectional promoters (see Figure 1; Guttman et al., 2009; Li and Ramchandran, 2010; Mattick and Rinn, 2015). Despite the diversities of lncRNAs, they share some common features, including: (1) most lncRNAs are transcribed by RNA polymerase II, spliced and modified with a 5′-cap and a poly-A tail, which makes them undistinguishable from protein-coding mRNAs; (2) they are poorly conserved at the sequence level, have a relatively low expression level and display a much more cell-tissue-specific pattern; and (3) they are generally regulated by transcription factors (Xiong et al., 2016). In addition, another subgroup of lncRNAs, the circular RNAs (circRNAs) have recently come into focus with the discovery of their pervasiveness and evolutionary conservation in mammalian and human cells (Jeck and Sharpless, 2014). Most circRNAs are generated during splicing either by spliceosomal machinery or by ribozymes I and II which thus splice out non-coding sequences from exons (exonic circRNAs), introns (intronic circRNAs), or a combination of introns and exons (exon-intron circRNAs; Abdelmohsen et al., 2015). CircRNAs can be differentiated from their linear counterparts by their adoption of a circular form and their lack of 5′ and 3′ ends (Vicens and Westhof, 2014).

### General Functions of LncRNAs

**Figure 2:**
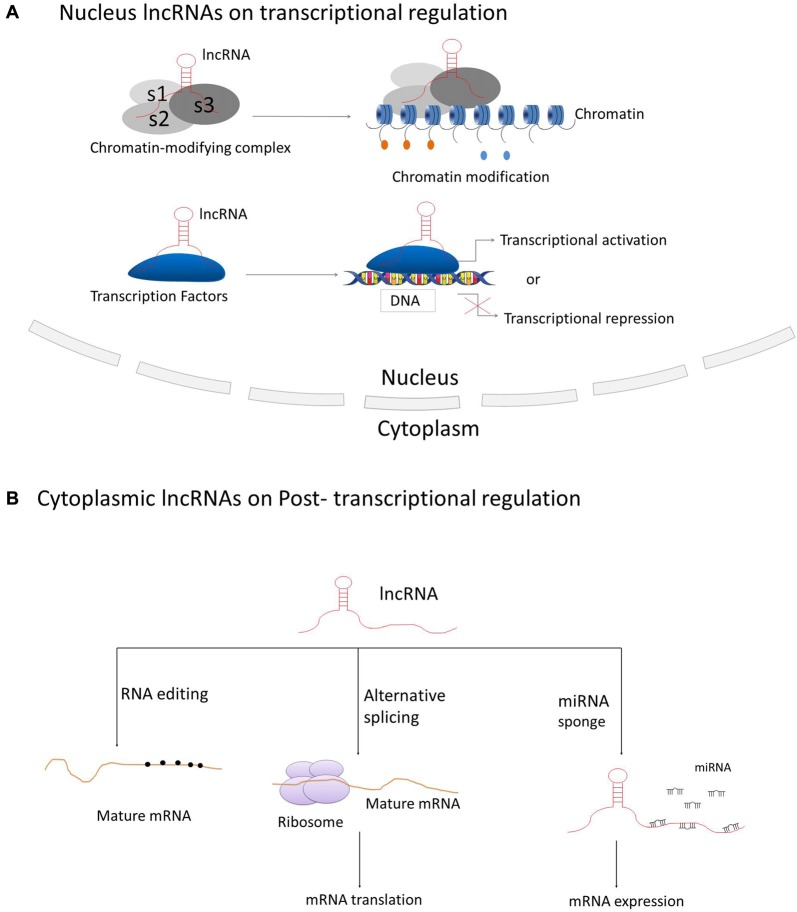
Principle mechanisms of lncRNAs on regulation of gene and genome activity. **(A)** LncRNAs located in the nucleus are basically functioning in transcriptional regulation through interacting with chromatin-modifying complexes or transcription factors; **(B)** Cytoplasmic lncRNAs are generally acting as regulators on RNA processing, such as RNA editing, alternative splicing and miRNA-mediated mRNA expression.

LncRNAs have been best described for their participation in regulating gene and genome activity at various levels (see Figure 2). LncRNAs can regulate the expression of nearby genes on the same allele *in cis*, or *in trans* to control genes at other genomic locations on different chromosomes, through which they can regulate gene expression at diverse levels, such as transcription, RNA processing and translation (Elling et al., 2016). The majority of lncRNAs are localized in the nucleus, in which they can fulfill their regulatory functions via acting as scaffolds for chromatin modifiers by interacting with chromatin-modifying complexes or as transcriptional co-regulators by binding to transcription factors (Rinn and Chang, 2012; Ulitsky and Bartel, 2013).

The best-known case of lncRNA that regulates transcription mediated through chromatin modification is *Xist*, a 17 kb lincRNA generated from the inactive X-chromosome (Clemson et al., 1996). It mediates the silencing of the inactive X-chromosome in human female cells through recruiting Polycomb Repressive Complex 2 (PRC2) by the Repeat A motif (RepA) on *Xist* and thus initiating chromosome-wide silencing via catalyzing Lysine 27 trimethylation on histone H3 (H3K27; Pinter et al., 2012; Jiang et al., 2013; Bergmann and Spector, 2014). *Hotair*, a 2.2 kb conserved lncRNA transcribed from the human HOXC locus on chromosome 12, is involved in repressing transcription *in trans* across the HOXD locus. *Hotair* was shown to physically interact with PRC2 to ensure the PRC2 occupancy and histone H3 lysine-27 trimethylation of HOXD locus (Rinn et al., 2007). The lncRNA *Braveheart*, prominently expressed in the mouse heart, can interact with Suz12, a subunit of PRC2 (Klattenhoff et al., 2013). The lncRNA *Fendrr* (Fetal-lethal developmental regulatory RNA), that is also related to cardiac development and heart function, can bind to PRC2 and WDR5, a member of the MLL histone methyl-transferase complex (Grote and Herrmann, 2013; Grote et al., 2013).

There are also cases that many lncRNAs interact with transcription factors. The definitive endoderm-associated lncRNA1 (*DEANR1*) is crucial for human endoderm differentiation via interaction and upregulation of the endoderm factor FOXA2 (Jiang et al., 2015). The *lnc-DC* (*Lnc dendritic cells*) is a lncRNA exclusively expressed in human conventional dendritic cells. It was revealed that *lnc-DC* can bind to the transcription factor signal transducer and activator of transcription 3 (STAT3) directly in the cytoplasm and induce its phosphorylation on Tyrosine-75 by inhibiting its binding to and dephosphorylation by SHP1, thereby leading to the activation of STAT3 on dendritic cell differentiation (Wang et al., 2014). In addition, the lncRNA breast cancer anti-estrogen resistance 4 (*BCAR4*), functioning in breast cancer metastasis, was discovered to directly interact with Smad nuclear-interacting protein 1 (SNIP1) and Serine/threonine-protein phosphatase one regulatory subunit 10 (PPP1R10 or PNUTS), thus activating phosphor-GLI2 dependent gene expression (Xing et al., 2014).

Cytoplasmic lncRNAs are also acting as modulators on post-transcriptional regulation of genes through various mechanisms during RNA processing, such as mRNA editing, alternative splicing and others. The antisense intronic lncRNA *prostate cancer antigen 3* (*PCA3*), acting as a dominant-negative oncogene, was demonstrated to interact with and down-regulate an as yet to be determined tumor suppressor gene *PRUNE2*, by the formation of *PRUNE2/PCA3* double-stranded RNAs that allow the adenosine deaminase to edit RNAs via adenosine to inosine editing (A-to-I editing; Salameh et al., 2015). Non-coding RNA activated by DNA damage (*NORAD*) is an abundant and highly conserved human lncRNA that acts as a multivalent binding platform for RNA binding proteins in the PUMILIO family in order to maintain genomic stability (Lee et al., 2016). Furthermore, the lncRNA-*asFGFR2* is an evolutionarily conserved nuclear antisense lncRNA that was generated from within the human FGFR2 locus. It was found to modulate the epithelial- specific alternative splicing of *FGFR2* by recruiting PRC2 and histone demethylase KDM2a in PNT2 cells (Gonzalez et al., 2015).

Recent studies also revealed certain lncRNAs acting as “miRNA sponges” that they have the potential to sponge and compete with miRNA target genes for the binding of miRNA response elements (MREs) to relieve miRNA-mediated target mRNA repression (Ebert and Sharp, 2010). The circRNA *ITCH* (*cir-ITCH*) was newly discovered to be functionally sponging miR-7, miR-17 and miR-214 and inducing the expression level of *ITCH*, which induced the ubiquitination and degradation of phosphorylated Dvl2 and thereby the inhibition of the Wnt/β-catenin pathway (Li et al., 2016). The lncRNA urothelial carcinoma-associated 1 (*UCA1*) was shown to work as an endogenous sponge that can down-regulate miR-216b expression by directly binding to miR-216b (Wang et al., 2015). The lncRNA human ovarian cancer-specific transcript 2 (*HOST2*) was verified to be a molecular sponge that can modulate the availability of miR let-7b (a potent tumor suppressor) and inhibit miR let-7b functions, thus post-transcriptionally suppressing target gene expressions (Gao et al., 2015).

## LncRNAs Play Crucial Roles in the CNS

### General Features of LncRNAs in Brain

Based on studies from the GENCODE project in 2012 that 10,000–50,000 lncRNAs in the human genome have been annotated to date, it was remarkably revealed that approximately 40% of lncRNAs (which is about 4000–20,000 lncRNAs) are tissue-specifically expressed in the brain (Derrien et al., 2012). This number is strikingly large in comparison to the number of protein-coding genes in human genome which is approximately 20,000–25,000 in general (Briggs et al., 2015). These brain-specific lncRNAs display the highest signals of evolutionary conservation in comparison with those expressed in other tissues. Studies also found that brain-expressed lncRNAs are enriched in predicted, conserved RNA structures and thus are more likely to possess conserved functions (Ponjavic et al., 2009). In addition, brain-expressed lncRNAs show greater brain region, temporal and spatial specificity than mRNAs. Numerous transcriptome analysis have revealed that various lncRNAs are expressed differentially over time and/or in those brain regions, such as cortex, cerebellum and hippocampus during development and adulthood (Lipovich et al., 2014; Kadakkuzha et al., 2015). LncRNAs are also found to be expressed in a more cell-type-specific manner than protein-coding genes. The transcriptome studies on cortical pyramidal neurons have uncovered 806 of 5195 lncRNAs have differential expression across neuronal types, of which, 55% of lncRNAs are annotated as cell-type signature cluster, 32% of lncRNAs are related to cell-type independent clusters, while around 10% of lncRNAs are found in the mixed cell-type clusters, indicating their role on the specification and maintenance of cell identity (Molyneaux et al., 2015). Another feature of brain-specific lncRNAs is their preferential location adjacent to brain-expressed protein-coding genes that are active in transcriptional modulation or in the nervous system development (Ponjavic et al., 2009). Transcriptome sequencing analysis in the corticogenesis displayed that most lncRNAs overlap with neurogenic genes and share with them a similar expression pattern, indicating that lncRNAs regulate corticogenesis through the tuning of the expression of nearby cell fate determinants (Aprea et al., 2013). Considering the complexity and heterogeneity of the mammalian CNS, the brain would be considered as the largest repertoire of lncRNAs in comparison to other somatic tissues and the tissue and cell-type specificity of these lncRNAs make them greatly contribute to cell fate, lineage specification and maintenance of cell identity during the development of the mammalian brain (Hart and Goff, 2016).

### Molecular Mechanisms of LncRNAs in CNS Development

**Figure 3:**
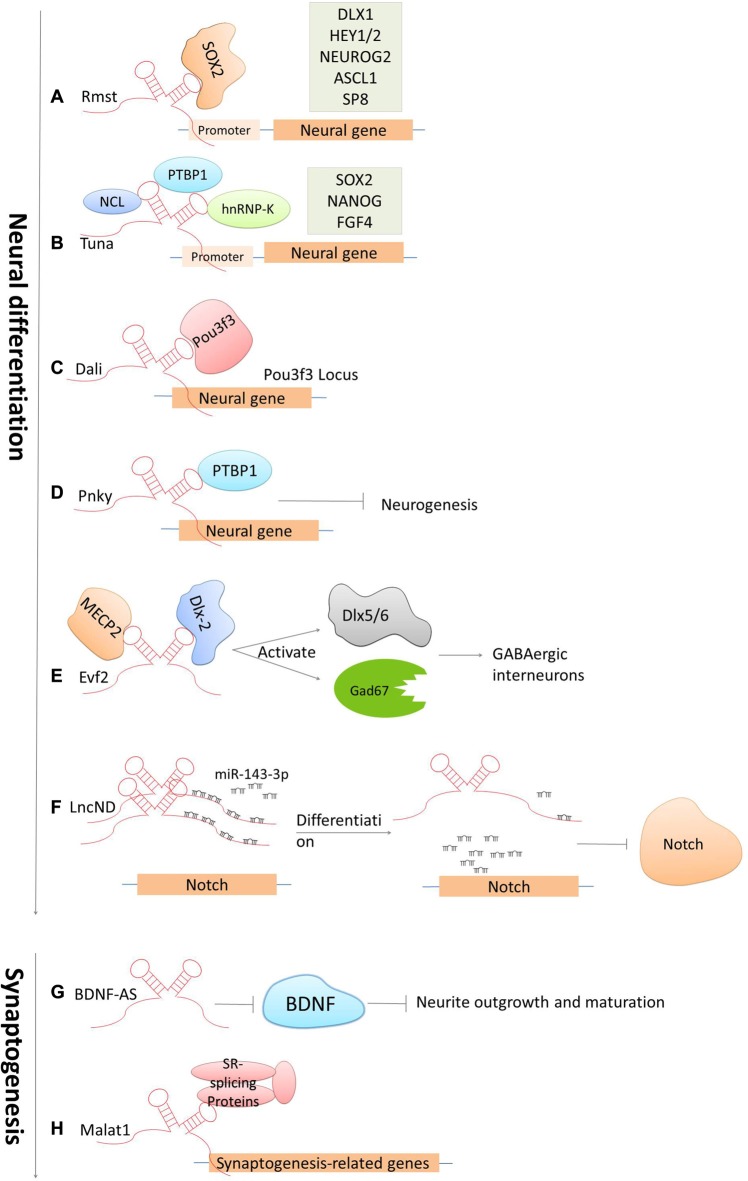
Cases of lncRNAs functioning in neuronal development. **(A–F)** LncRNAs function in early neural commitment through the recruitment of the transcriptional machinery to regulate neural-related gene expressions (*Rmst, Tuna, Dali and Pnky*). *Evf2* is particularly acting in regulating GABAergic interneuron specification. *LncND* functions in regulating Notch signaling pathways via sequestering miR143-3p. **(G–H)** LncRNAs function in late developmental processes, such as neurite outgrowth and maturation mediated by *BDNF-AS*, synapse function by *Malat1*.

The development of the CNS is a complicated and highly stereotyped process that requires elaborate spatiotemporal regulation of stem/progenitor cell proliferation and differentiation. LncRNAs have been demonstrated to play indispensable roles in CNS development from early neural differentiation to late-stage synaptogenesis (see Figure 3; Briggs et al., 2015).

Accumulated studies have confirmed that dozens of lncRNAs are identified to be functional in establishing pluripotency or driving neural lineage entry in the widely used *in vitro* model systems—mouse embryonic stem cells (ESCs; Guttman et al., 2011; Ng et al., 2012). These lncRNAs are functional at various stages along the progression from pluripotent cells in the early embryo to the terminal cell types in the mature mammalian brain. The regulatory mechanisms are generally based on modulating these lncRNAs by canonical pluripotency transcription factors, which in turn perform their regulatory effects by directing transcription factors or chromatin remodeling complexes to specific lineage-specifying genes. For examples, the lncRNA Rhabdomyosarcoma 2-associated transcript (*Rmst*), specifically expressed in the brain and regulated by the transcription repressor REST, was found to modulate neural differentiation *in vitro*. It was shown that *Rmst* interacts with SOX2 to co-regulate a large pool of downstream genes (such as DLX1, HEY2 and SP8) implicated in neurogenesis. The absence of *Rmst* can block the exit from the ESC state and the initiation of neural differentiation (Ng et al., 2013). *Tuna*, is also a lncRNA that regulates neuronal gene expression in a similar mechanism as *Rmst*. *Tuna* interacts with three RNA-binding proteins (NCL, PTBP1 and hnRNP-K) and together target promoter regions of neural genes in differentiating mouse ESCs. The knockdown of *Tuna* or any of the three RNA-binding proteins can sufficiently suppress neural differentiation (Lin et al., 2014). This is also conserved from relatively related vertebrates, mice and zebrafish, indicating that the neural lineage commitment driven by lncRNAs displays the highly evolutionary conservation (Briggs et al., 2015). Furthermore, the lncRNA *Dali*, transcribed downstream of the transcription factor Pou3f3, was shown to drive the expression of an essential gene involved in neuronal differentiation in neuroblastoma cells. *Dali* can regulate transcription of the *Pou3f3* locus locally and physically interact with Pou3f3 protein to regulate the expression of neural differentiation genes distally (Chalei et al., 2014). *Paupar*, a chromatin-associated intergenic lncRNA expressed in the CNS, is divergently transcribed from a locus upstream from the gene encoding the transcription factor *Pax6*. It was shown that the knockdown of *Paupar* destroys the normal cell cycle profile of neuroblastoma cells, thus enhancing neuronal differentiation. The function of *Paupar* is performed via locally interacting with and transcriptionally regulating *Pax6*, as well as via distally controlling neural gene expression on a large scale (such as *SOX2*, *HES1* and *EVF2*), which in part requires physical association with Pax6 protein (Vance et al., 2014). Besides that, one of the latest studies has identified an lncRNA, termed *LncND* (Neurodevelopment) that harbors 16 miRNA response elements for miR-143-3p in primates. It performs its role in neuronal development by sequestering miR-143-3p, and in doing so, modulates Notch signaling pathways via regulating expression of Notch receptors, *NOTCH-1* and *NOTCH-2*. Also, *NOTCH-1* and *NOTCH-2* show the same expression pattern as *LncND* in early neurogenesis process when Notch expression is indispensable. While, later in neural differentiation process, reduction of *NOTCH* expression is followed by *LncND*, leading to the release of miR-143-3p and the decrease of Notch signaling specifically in *LncND*-expressing cells (Rani et al., 2016). Interestingly, knocking down *LncND* in neuroblastoma cells can inhibit *NOTCH-1* and *NOTCH-2* and make cells differentiate to neurons, which show similar phenotypes as what can be observed by miR-143-3p overexpression. These findings suggested a role of *LncND* in miR-mediated regulation of Notch signaling to sustain the neural progenitor pool during cerebral cortex expansion in primates (Rani et al., 2016).

There are also several important lncRNAs being identified to control stem cell turnover and the specification of particular lineages in the embryonic mouse brain *in vivo*. For example, *Evf2*, the firstly identified nervous system-specific lncRNA, has been shown to have a significant regulatory role in neural development. Depletion in *EVF2* can cause imbalance of the excitatory to inhibitory neurons in the postnatal hippocampus and dentate gyrus. This imbalance is caused by the failure of GABAergic interneuron specification (Bond et al., 2009). It was revealed that *Evf2* recruits DLX and methyl CpG-binding protein (MECP2) to modulate the transcriptional activity of two transcription factors Dlx5/6 and the glutamate decarboxylase 67 (Gad67, which is required for the conversion of glutamate to GABA), and thereby regulates the gene expression of GABAergic interneurons in the developing mouse forebrain (Feng et al., 2006; Bond et al., 2009). *Pnky* is an evolutionally conserved and neural-specific lncRNA that modulates neurogenesis of neural stem cells (NSC) in the embryonic and postnatal brain. The Ramos AD group (Ramos et al., 2013) has unveiled that knockdown of *Pnky* can increase neuronal differentiation and deplete the NSC population in the embryotic mouse cortex. In addition, it was shown that *Pnky* interacts with PTBP1, a splicing regulator expressed in NSCs that represses the inclusion of neural exons in non-neural cells. Knockdown of *PTBP1* also reinforce neurogenesis, indicating that *Pnky* and PTBP1 function similarly to modulate the alternative splicing of a group of transcripts that are involved with cellular phenotypes (Aprea and Calegari, 2015; Ramos et al., 2015). Together, these studies indicate that lncRNAs regulate cell-fate determination and progenitor cell turnover during neural development both *in vitro* and *in vivo*.

Another key process during CNS development is the late-stage synaptogenesis. Accordingly, several lncRNAs have been discovered to make great contributions to this process (Briggs et al., 2015). *Malat1* is a well-known lncRNA that have been implicated in the regulation of neurite elaboration. *Malat1* is expressed in multiple tissues but is highly abundant in neurons. It was revealed that *Malat1* can actively recruit SR-family splicing proteins to transcription sites in order to regulate synaptogenesis-related gene expression. The knockdown of *MALAT1* leads to a decreased level of synaptic density; while in contrast, overexpression can reciprocally increase synaptic density (Bernard et al., 2010). Primate-specific *BC200* RNA (Brain cytoplasmic RNA, 200 nt) and its rodent counterpart *BC1* are evolutionarily conserved lncRNAs that have been firstly identified to be expressed in both developing and adult nervous systems (Muslimov et al., 1997). Studies later reported that *BC200/BC1* is functional in the neuronal excitation-repression equilibria via protein-synthesis-dependent implementation (Zhong et al., 2009). In addition, several antisense lncRNAs have been recently presented as core protein regulators, for example Brain-derived neurotrophic factor (BDNF), glial-derived neurotrophic factor (GDNF) and ephrin receptor B2 (EPHB2) that control neurite elaboration. The expression of *BDNF* is repressed by its antisense lncRNA *BDNF-AS*. The inhibition of *BDNF-AS* can release and induce the expression of *BDNF* by 2- to 7-folds, which is in line with the reduction of EZH2 recruitment and an alternation of the chromatin state at the *BDNF* locus. This correspondingly induces neuronal outgrowth, differentiation, survival and proliferation both *in vitro* and *in vivo* (Modarresi et al., 2012). These observations, along with the current understanding of lncRNA mechanisms of action, imply that lncRNAs have a critical role in regulating neural gene expression and brain development.

### Expression Profiles of LncRNAs in Major Cell Types of the CNS

LncRNAs have been proposed to play diverse roles in the CNS, such as in neural differentiation, in synaptogenesis and others. As a result, the expression profiles of lncRNAs in the CNS were recently studied. The CNS has prominent cellular diversities owning hundreds of distinct cell types. In particular, neurons and neuroglia cells (mainly including astrocytes and oligodendrocytes) are the major cell types. A recent study has successfully isolated nuclear RNAs from different CNS cell types (neurons, astrocytes and oligodendrocytes) and compared both protein-coding and noncoding nuclear transcriptome profiles in these three cell types (Reddy et al., 2017). For non-coding transcriptome, it was revealed that approximately 300 transcripts at a level of >5 CPM (counts per million) can be observed in one of the three different cell types, and the majority is transcribed at the level of >1 CPM. Several other highly expressed transcripts are also found in all three cell types, such as *Xist* and its regulators *Ftx* and *Jpx* (Tian et al., 2010; Chureau et al., 2011; Reddy et al., 2017). Nevertheless, 169 lincRNAs were shown to have a >10-fold difference in one of the pairwise comparisons, such as *Mirg* and other adjacent maternally expressed lncRNAs (*Meg3* and *Rian*) that are found to be highly enriched in neuronal nuclei. *Mirg* was found to be precisely localized within bright subnuclear puncta in neurons; the expression of *Meg3* was shown as neuronal selective that it was enhanced in gray matter where neurons are found but depleted from white matter areas where only glia are found; and *Rian* was found to have similar expression pattern as *Meg3* (Zhang et al., 2003; Balik et al., 2013; Reddy et al., 2017). In astrocytes, various transcripts were found but most of them do not possess known functions. Whereas, the lncRNA *Rmst*, a key co-regulator of neurogenesis with the SOX2 transcription factor, is an exception that it has been shown to be expressed robustly in the nuclei of astrocytes (Ng et al., 2013; Reddy et al., 2017). Meanwhile, it was shown that there are at least 16 lncRNAs with >5 CPM found in oligodendrocytes, such as *Neat1* and *DLeu2*. Notably, although *Neat1* was shown to have the highest expression level in oligodendrocytes, it was also present in astrocytes (Klein et al., 2010; Reddy et al., 2017). These data demonstrated different lncRNA expression patterns in three major cell types in the brain, providing a crucial clue for evaluating functions of various lncRNAs in the brain and CNS-associated disorders.

## LncRNAs Tightly Associate with Neurodegenerative Diseases

The importance of lncRNAs in the brain has been asserted by their association with various brain functions, including the maintenance of pluriotency, neuroectodermal differentiation, neuron-glial cell fate determination, synaptogenesis and so on (Roberts et al., 2014). Considering that, it is not surprising that dysregulation or mutation of lncRNAs is tightly related to various neurological disorders. Genome-wide association studies and comparative transcriptome analysis have implied that lncRNAs are involved in a variety of neurological disorders, such as psychiatric disorders, neurodegenerative diseases (like Alzheimer’s disease-AD, Parkinson disease-PD, Huntington disease-HD, Frontotemporal lobar degeneration-FTLD and Amyotrophic lateral sclerosis-ALS, etc.), and others. Similarly, as described above, lncRNAs contribute to these diseases in diverse ways, from the regulation of transcription to the modulation of RNA processing and translation (see Table 1). Here, we briefly exemplified several studied cases of lncRNAs that have been identified to be associated with neurodegenerative diseases.

**Table 1:** Cases of neurodegenerative diseases-associated lncRNAs.

Regulatory functions	lncRNAs	Targets	Regulating	Related pathologies	In which regions/cells	Diseases involved	References
Transcription	*HTT-AS*	HTT	Downregulated	Regulates HTT expression	Frontal cortex	HD	Chung et al. (2011)
Transcription	*C9ORF72*	Yet to be determined	Upregulated	Containing the G_4_C_2_ expansion	Frontal and motor cortex, hippocampus, spinal cord neurons	ALS/FTLD	Zu et al. (2013)
Transcription	*NEAT1–2*	TDP-43; FUS/TLS	Upregulated	Regulate the transcription or transcript stability of lncRNAs	Spinal motor neuron	ALS/FTLD	Nishimoto et al. (2013)
Transcription	*GDNFOS1*	GDNF	Upregulated		Middle temporal gyrus	AD	Airavaara et al. (2011)
mRNA stability	*BACE1-AS*	BACE1	Upregulated	Induces Aβ40 and Aβ42 production	Prefrontal cortex, striatum, cerebellum, hippocampus anterior & posterior,	AD	Faghihi et al. (2008); Modarresi et al. (2011)
Alternative splicing	*17A*	GPR51	Upregulated	Abolishes GABA B2 intracellular signaling and increase Aβ secretion	Cerebral Cortex	AD	Massone et al. (2011)
Post-transcriptional	*NAT-Rad18*	Rad18	Upregulated	Affects ability of neuron and their apoptosis susceptibility	Cortical neuron	AD	Parenti et al. (2007); Wu et al. (2013)
miRNA sponge	*ciRS-7*	UBE2A	Upregulated	Regulated AD-associated targets	Hippocampal CA1	Sporadic AD	Cogswell et al. (2008); Hansen et al. (2013); Lukiw (2013)
Translation	*BC200*	SYNCRIP	Upregulated	Regulates dendritic mislocalization	Brodmann’s area 9	AD	Muddashetty et al. (2002); Mus et al. (2007); Duning et al. (2008)
Translation	*Uchl1-AS*	Uchl1	Downregulated	Regulates protein synthesis of Uchl1	Dopaminergic neurons	PD	Carrieri et al. (2012)

*Huntingtin (HTT)* is an essential gene for HD, a CAG trinucleotide repeat expansion in exon 1, and the main cause of HD. The *HTT antisense* (*HTT-AS*), a natural antisense transcript at the HD repeat locus containing the repeat tract, was identified and characterized with 5′ capped, poly A tailed, three exons maintained and alternatively being spliced into *HTTAS-v1* (exons 1 and 3) and *HTTAS-v2* (exons 2 and 3). Cell studies revealed that the overexpression of *HTTAS-v1* specifically decreases endogenous HTT transcript levels, whereas the siRNA knockdown of *HTTAS-v1* induces *HTT* transcript levels. What’s more, *HTTAS-v1* expression was found to be downregulated in frontal cortex of HD patients, strongly suggesting the existence of a gene antisense to *HTT* acting as a regulator for its own expression and its contribution to the development of HD (Chung et al., 2011).

Beta-secretase 1 (BACE1) is the key enzyme that produces β-amyloid peptide (Aβ) which aggregates and forms into amyloid plaques as a main pathological hallmark of AD. Recent studies have identified a conserved noncoding antisense transcript of BACE1, *BACE1-AS*, that regulates *BACE1* mRNA and subsequently BACE1 protein expression both *in vivo* and *in vitro*. Studies have highlighted that the knockdown of *BACE1-AS* can reduce BACE1 levels, as well as Aβ1–40 and Aβ1–42 production correspondingly. Exposure to various cell stressors (including Aβ1–42) that have been implicated in the pathogenesis of AD, was found to induce *BACE1-AS* levels. This induction is led by the formation of a RNA duplex with *BACE1* mRNA, which in turn increases *BACE1* mRNA stability and BACE1 protein, and consequently generates additional Aβ1–42 through a post-transcriptional free-forward mechanism (Faghihi et al., 2008). Furthermore, it was found that increased *BACE1-AS* levels exist in various brain regions in subjects with AD in comparison to control individuals, indicating the possibility of *BACE1-AS* being a potential biomarker of AD (Faghihi et al., 2008; Modarresi et al., 2011).

Recently, *17A* was described as an antisense transcript of the human G-protein-coupled receptor 51 gene (GPR51, GABA B2 receptor) that is RNA polymerase III-dependent and embedded in the GPR5. In neuroblastoma cells, the stable expression of *17A* can promote the synthesis of an alternative splicing isoform for GABA R2, resulting in the elimination of GABA B2 intracellular signaling and the enhancement of Aβ secretion and the ratio of Aβ42/Aβ40. Furthermore, *17A* is expressed in the human brain and upregulated in cerebral tissues derived from AD patients, indicating its potential contribution to the development of AD (Massone et al., 2011).

*NAT-Rad18*, a natural antisense transcript against *Rad18* (a gene encoding DNA repair protein), was investigated and considered to play potential roles in the DNA damage repair system in AD. RNA quantitative and immunohistochemistry analysis revealed that *NAT-Rad18* is widely distributed in the adult rat brain, but with high levels in the cerebellum, brainstem and cortex where neurons are specifically expressed. Upon Aβ-induced apoptosis in cortical neurons, the expression of *NAT-Rad18* was shown to be up-regulated, whereas *Rad18* was post-transcriptionally down-regulated. This observation suggested NAT-Rad18 might reduce the ability of neurons and increase their apoptosis susceptibility via the post-transcriptional modulation of *Rad18* to reduce their response to DNA damage stress (Parenti et al., 2007; Wu et al., 2013).

*GDNFOS*, transcribed from the opposite strand of GDNF, was demonstrated to be associated with neurodegenerative diseases, like AD. *GDNFOS* contains four exons that therefore are spliced into different isoforms, including *GDNFOS1/2* acting as *lncRNAs* and *GDNFOS3* encoding a protein of 105 amino acids (Airavaara et al., 2011). It was revealed that the mature GDNF peptide was reduced while the transcript *GDNFOS1* upregulated in the postmortem middle temporal gyrus of patients suffering from AD when compared with those of normal controls, indicating the dysregulation of *GDNF* and *GDNFOS* might further implicate in other human brain diseases (Airavaara et al., 2011).

One brain-specific circRNA, *ciRS-7* (circular RNA sponge for miR-7), also known as *CDR1as*, is transcribed antisense to the cerebellar degeneration-related protein 1 transcript (*CDR1*) that is highly expressed (even more than the sense transcript) in the mouse and human CNS. The *ciRS-7* functions as a miR-7 sponge that strongly quenches miR-7 activity, thus causing induced levels of miR-7 targets. Recent studies also indicated an endogenous interaction between *ciRS-7* and miR-7 based on the observation of the co-expression of *ciRS-7* and miR-7 in the mouse brain (Hansen et al., 2013; Memczak et al., 2013). The *ciRS-7* has been identified to be related to the sporadic AD, that dysregulation of *ciRS-7* was evidenced in the hippocampal CA1 region of the sporadic AD (Lukiw, 2013). The *ciRS-7* deficiency was expected to induce ambient miR-7 levels in AD-affected brain cells, which is probably responsible for down-regulating AD-associated targets, such as, the ubiquitin protein ligase A (UBE2A; Cogswell et al., 2008; Lukiw, 2013).

*BC200* RNA is tightly related to AD development and its expression has been demonstrated to be substantially up-regulated in tested AD brain tissues (Brodmann’s area 9) in comparison to that in age-matched normal brain samples (Mus et al., 2007). Further analysis also demonstrated that the increase in levels of *BC200* RNA only occurs in specific regions of the AD-brain and is accompanied by changes in *BC200* RNA neuronal distribution, including dendritic mislocalization and gradual accumulation of *BC200* RNA to the perikaryon (Mus et al., 2007). The role of *BC200* RNA in regulating gene expression at translational level during the development of AD has been reported in many studies as being through mechanisms of interaction with many different proteins. It was shown that *BC200* RNA interacts with the human synaptotagmin-binding cytoplasmic RNA interacting protein (SYNCRIP), a component of large mRNA transport granules in neurons and functioning in local protein synthesis at post-synaptic sites, through mediation by the N-terminal RNA recognition motifs and the central A-rich region of *BC200* RNA (Duning et al., 2008). In addition, the polyA-binding protein (PABP1), a regulator of translation initiation, was also identified to bind to *BC200* RNA mediated by its central A-rich region, leading to the hypothesis that *BC200* RNA is associated with protein translation in neuronal dendrites (Muddashetty et al., 2002). The interactions between *BC200* RNA and proteins involved in local protein synthesis in neurons indicated the important role of *BC200* RNA in AD pathology.

*The Uchl1-AS* is a nuclear-enriched lncRNA that is transcribed antisense to the mouse ubiquitin carboxy-terminal hydrolase L1 (Uchl1). Uchl1 is a neuron-restricted protein acting as a de-ubiquitinating enzyme or a monoubiquitin stabilizer. *UCHL1* gene mutations have been discovered to be related to familial PD, and the oxidative inactivation of Uchl1 protein has been reported in PD and AD brains (Choi et al., 2004; Barrachina et al., 2006). *Uchl1-AS* can increase the protein synthesis of *UCHL1* at post-transcriptional level, which depends on the combined activities of two domains, the 5′ antisense region that provides specificity for the sense target gene and the embedded repetitive SINEB2 element (short interspersed nuclear element of B3 subclass) that confers the protein synthesis activation domain (Nishihara et al., 2006; Carrieri et al., 2012). In addition, the activity of *Uchl1-AS* is under the control of signaling pathways. Uchl1 mRNA is predominantly localized in the cytoplasm while *Uchl1-AS* is abundant in the nucleus of dopaminergic neurons. Intriguingly, the mTOR inhibitor-Rapamycin treatment resulted in the induction of Uchl1 protein by association of shuttling *Uchl1-AS* from the nucleus to the cytoplasm, indicating the interplay among Uchl1-ncRNA-mTOR might be crucial for the development of PD (Carrieri et al., 2012; Vučičevič et al., 2014).

*C9ORF72*, chromosome 9 ORF72, contains a hexanucleotide (GGGGCC, G_4_C_2_) repeat expansion in its non-coding region, which was found as the causative mutation for both ALS and FTLD (DeJesus-Hernandez et al., 2011). The *C9ORF72* expansion mutation can be transcribed bidirectionally that produces unexpected proteins via repeat-associated non-ATG (RAN) translational mechanism. Recent discovery found that antisense *C9ORF72* transcripts containing the G_4_C_2_ expansion are increased in ALS patients’ brains (Zu et al., 2013), indicating the vital role of antisense *C9ORF72* transcripts on fundamental pathologies of ALS/FTLD.

In addition, the lncRNA nuclear-enriched abundant transcript 1–2 (*NEAT1-2*), containing nuclear bodies named as “paraspeckles”, has also been shown to be associated with ALS/FTLD. TAR DNA binding protein 43 (TDP-43) inclusions or that are fused in sarcoma/translocated in liposarcoma (FUS/TLS) are displayed and characterized as the major pathology of ALS/FTLD (Nishimoto et al., 2010). Studies revealed that TDP-43 and FUS/TLS are enriched in paraspeckles and bound to NEAT1-2 directly. Furthermore, the expression of *NEAT1-2* was shown to be enriched and the specific assembly of NEAT1-2 as paraspeckles be formed in spinal motor neurons at an early stage of the ALS pathogenesis (Nishimoto et al., 2013; Lourenco et al., 2015). These suggested *NEAT1-2* might be functional in modulation of ALS-associated RNA-binding proteins at the early stage of ALS.

## Challenges and Perspectives

Along with intensive studies of lncRNAs, there are still challenges for our understanding of lncRNAs. On one side, most investigations of lncRNA are limited to single studies, and some of them are only an *in vitro* study. Kohtz (2014) has stated that the roles of lncRNAs in cell lines can be distinguished with the ones examined in animal models (*in vivo*). Taking *Evf2* as an example, *Evf2* cell line assays indicated the *in trans* mechanism of lncRNA functioning as activation enhancers, whereas the knockdown of *Evf2* in *Evf2^TS/TS^* mice associated lncRNA with repression *in cis* (Feng et al., 2006; Bond et al., 2009). On the other side, some lncRNAs are only studied in mouse models with genomic deletions. With respect to the characters of lncRNAs that are located in the nucleus and chromatin-associated that might be *cis*-acting transcriptional regulators, or in cytoplasm that might be predicted to act *in trans*, the phenotypes produced by deletion of an entire genomic locus apparently cannot be equivalently the same as the loss of lncRNA *per se* or as the associated loss of other overlapping DNA regulatory elements (Bassett et al., 2014). In these cases, it will be difficult to distinguish from the effects caused by loss of a lncRNA transcript to the general effects caused by the loss of the genomic region itself (Kohtz, 2014; Aprea and Calegari, 2015). Considering these challenges, researchers have proposed the importance of optimizing currently employed techniques along with developing advanced ones as tools to help differentiating between the influences of lncRNAs when they act as molecular species compared to when they act as gene regulatory elements. For example, fluorescent *in situ* hybridization (FISH) has been widely applied for the analysis of lncRNA localization in tissues and subcellular levels (Chakraborty et al., 2012). Also, a series of interaction assays for the identification of protein or nucleic acids with lncRNAs are applied, such as protein-RNA (crosslinking immunoprecipitation, CLIP), RNA-RNA (crosslinking analysis of synthetic hybrids, CLASH) or RNA-DNA (capture hybridization analysis of RNA targets, CHART; Helwak et al., 2013; Huppertz et al., 2014; Vance and Ponting, 2014). Apart from these technical updates, the Clustered regularly interspersed palindromic repeats (CRISPR/Cas9) system, functioning as a mechanistic tool, is popularly used for targeted genome engineering (Cheng et al., 2013; Bassett et al., 2014). With regards to this, the landscape of lncRNAs with proven functions in various biological processes have been substantially increasing and this range is expected to be further expanding in the coming years.

The brain is the organ where more lncRNAs are abundantly expressed, comprising the highest proportion of brain-specific lncRNAs (Derrien et al., 2012). Although limited numbers of lncRNAs have been identified to be associated with the complexity of the brain functions, lncRNAs are involved in brain functions in both a normal and diseased state. It is implied that brain-specific lncRNAs may be innovatively evolved, involved in human brain development and related to neurodegenerative diseases (Vučičevič et al., 2014). Based on studies of lncRNAs functioning in brain development and pathophysiology of neurological disorders, the definition criteria include: (1) genetic variation in lncRNA genes leads to disease and influences susceptibility; (2) epigenetic deregulation of lncRNAs is associated with disease; (3) genomic context binds lncRNAs to disease-related genes and pathways; and (4) lncRNAs are interconnected with known pathogenic mechanisms (Qureshi and Mehler, 2013). However, it is worth noting that phenomena, like some lncRNAs either might show different expression levels in healthy brains compared to diseased brains, or they might interact with certain proteins that function in brain disorders, could be a result of multiple reasons that are not related to the disease or caused by unspecific side effects (Vučičevič et al., 2014). Apart from studies of lncRNA functions in cell lines that would reveal basic molecular mechanisms of lncRNAs, the generation of lncRNAs-knockdown or mutation in mouse models would be more effective to study the functional relevance of lncRNAs during brain development and in the physiological conditions of neurodegenerative disease.

In fact, as proposed by many studies, more and more attention has been given to lncRNAs as being disease biomarkers or potential targets for therapeutic strategies. Indeed, several commercial entities (e.g., OPKO-CURNA and RaNA therapeutics), which target lncRNAs have been developed to design and develop oligoneucleotide therapeutics for the treatment of CNS-related neurological disorders (Qureshi and Mehler, 2013). However, due to the large size of many lncRNAs, it became evident that the crossing of lncRNAs through the blood-brain barrier (BBB) will be a significant issue to be considered. Exosomes, 20–100 nm membrane nanovesicles of endocytic origin secreted by most cell types *in vivo* and *in vitro*, are shown to be natural carriers of functional RNAs (such as mRNA, miRNA, rRNA and lncRNAs) and proteins (Raposo et al., 1996; Valadi et al., 2007). Exosomes can also transfer these genetic materials between cells and subsequently modulate the functions of targeted cells. These characteristics indicate their important role in communications between cells, and therefore make them great potentials for therapeutic delivery (Kawikova and Askenase, 2015; Barile and Vassalli, 2017). Moreover, exosomes were found to have an impact on the pathophysiology of the brain due to the fact that they can also be released by CNS cells (Faure et al., 2006; Kawikova and Askenase, 2015). As suggested, utilization of exosomes as delivery cargo would be an efficient strategy for helping to bypass the BBB (Lakhal and Wood, 2011; Katakowski et al., 2013). Hereby, further investigation on the role of lncRNAs will provide a better understanding of how the brain functions and how diseases develop, and lead to greater insights into further therapeutic development for neurodegenerative diseases based on manipulations of lncRNA functions.

## Author Contributions

ZQ and HQ organized the article and ZQ drafted the manuscript; ZQ and DZ designed the pictures and prepared the draft; ZQ and HQ approved the final version.

## Conflict of Interest Statement

The authors declare that the research was conducted in the absence of any commercial or financial relationships that could be construed as a potential conflict of interest.
